# Duhamel and transanal endorectal pull-throughs for Hirschsprung disease: a Bayesian network meta-analysis

**DOI:** 10.1186/s12893-024-02416-0

**Published:** 2024-05-03

**Authors:** Dong Sun, Xintao Zhang, Qiongqian Xu, Yang Li, Qiangye Zhang, Dongming Wang, Weijing Mu, Peimin Hou, Aiwu Li

**Affiliations:** Department of Pediatric Surgery, Qilu Hospital, Shandong University, No.107 Wenhua West Road, Lixia District, Jinan, 250012 Shandong China

**Keywords:** Duhamel, Transanal endorectal pull-through, Hirschsprung disease, Bowel function, Network meta-analysis

## Abstract

**Background:**

To comprehensively compare the effects of open Duhamel (OD), laparoscopic-assisted Duhamel (LD), transanal endorectal pull-through (TEPT), and laparoscopic-assisted endorectal pull-through (LEPT) in Hirschsprung disease.

**Methods:**

PubMed, Embase, Cochrane Library, Web of Science, CNKI, WanFang, and VIP were comprehensively searched up to August 4, 2022. The outcomes were operation-related indicators and complication-related indicators. The Grading of Recommendations Assessment, Development and Evaluation (GRADE) approach was used to evaluate the quality of evidence. Network plots, forest plots, league tables and rank probabilities were drawn for all outcomes. For measurement data, weighted mean differences (WMDs) and 95% credibility intervals (CrIs) were reported; for enumeration data, relative risks (RRs) and 95%CrIs were calculated.

**Results:**

Sixty-two studies of 4781 patients were included, with 2039 TEPT patients, 1669 LEPT patients, 951 OD patients and 122 LD patients. Intraoperative blood loss in the OD group was more than that in the LEPT group (pooled WMD = 44.00, 95%CrI: 27.33, 60.94). Patients lost more blood during TEPT versus LEPT (pooled WMD = 13.08, 95%CrI: 1.80, 24.30). In terms of intraoperative blood loss, LEPT was most likely to be the optimal procedure (79.76%). Patients undergoing OD had significantly longer gastrointestinal function recovery time, as compared with those undergoing LEPT (pooled WMD = 30.39, 95%CrI: 16.08, 44.94). The TEPT group had significantly longer gastrointestinal function recovery time than the LEPT group (pooled WMD = 11.49, 95%CrI: 0.96, 22.05). LEPT was most likely to be the best operation regarding gastrointestinal function recovery time (98.28%). Longer hospital stay was observed in patients with OD versus LEPT (pooled WMD = 5.24, 95%CrI: 2.98, 7.47). Hospital stay in the TEPT group was significantly longer than that in the LEPT group (pooled WMD = 1.99, 95%CrI: 0.37, 3.58). LEPT had the highest possibility to be the most effective operation with respect to hospital stay. The significantly reduced incidence of complications was found in the LEPT group versus the LD group (pooled RR = 0.24, 95%CrI: 0.12, 0.48). Compared with LEPT, OD was associated with a significantly increased incidence of complications (pooled RR = 5.10, 95%CrI: 3.48, 7.45). Patients undergoing TEPT had a significantly greater incidence of complications than those undergoing LEPT (pooled RR = 1.98, 95%CrI: 1.63, 2.42). For complications, LEPT is most likely to have the best effect (99.99%). Compared with the LEPT group, the OD group had a significantly increased incidence of anastomotic leakage (pooled RR = 5.35, 95%CrI: 1.45, 27.68). LEPT had the highest likelihood to be the best operation regarding anastomotic leakage (63.57%). The incidence of infection in the OD group was significantly higher than that in the LEPT group (pooled RR = 4.52, 95%CrI: 2.45, 8.84). The TEPT group had a significantly increased incidence of infection than the LEPT group (pooled RR = 1.87, 95%CrI: 1.13, 3.18). LEPT is most likely to be the best operation concerning infection (66.32%). Compared with LEPT, OD was associated with a significantly higher incidence of soiling (pooled RR = 1.91, 95%CrI: 1.16, 3.17). Patients with LEPT had the greatest likelihood not to develop soiling (86.16%). In contrast to LD, LEPT was significantly more effective in reducing the incidence of constipation (pooled RR = 0.39, 95%CrI: 0.15, 0.97). LEPT was most likely not to result in constipation (97.81%). LEPT was associated with a significantly lower incidence of Hirschprung-associated enterocolitis (HAEC) than LD (pooled RR = 0.34, 95%CrI: 0.13, 0.85). The OD group had a significantly higher incidence of HAEC than the LEPT group (pooled RR = 2.29, 95%CrI: 1.31, 4.0). The incidence of HAEC was significantly greater in the TEPT group versus the LEPT group (pooled RR = 1.74, 95%CrI: 1.24, 2.45). LEPT was most likely to be the optimal operation in terms of HAEC (98.76%).

**Conclusion:**

LEPT may be a superior operation to OD, LD and TEPT in improving operation condition and complications, which might serve as a reference for Hirschsprung disease treatment.

**Supplementary Information:**

The online version contains supplementary material available at 10.1186/s12893-024-02416-0.

## Background

Hirschsprung disease is a congenital neurocristopathy, resulted from the migration, proliferation, differentiation, and survival defects of neural crest cells, bringing about intestinal aganglionosis [1, 2]. This disease is common in children, with an incidence rate ranging from 1/5000 to 1/2000, and it causes continuous intestinal spasm, fecal deposition in the proximal colon, hypertrophy, and expansion of the proximal colon, and then constipation, malnutrition, colitis, and other problems [3, 4]. Various operations have been proposed to treat Hirschsprung disease, with most cases having pull-through procedures. The purpose of a pull-through procedure is to remove the aganglionic colon, bring normally innervated bowel to the anus and preserve anal sphincter function [5, 6].

The Duhamel pull-through and the endorectal pull-through procedures are commonly used for the treatment of Hirschsprung disease [7]. The Duhamel technique involves the preservation of the native rectum and longitudinal anastomosis between the ganglionic colon and rectum [5]. In 1998, a modified single-staged Soave procedure was described [8], defined as the transanal endorectal pull-through (TEPT), with mobilization of the aganglionic colonic segments and stretching of the anal sphincters [9]. This technique owns the advantages of short hospital stay, less pain, and a low complication rate [10, 11]. Nevertheless, there is great concern about long-term anorectal function, including soiling and constipation [12, 13]. Both the TEPT and Duhamel techniques can be performed with the aid of laparoscopy, which leads to less trauma, blood loss, constipation, soiling, and intestinal adhesion [14–18]. Many studies showed the advantage of laparoscopic method over the open pull-through, while there were studies indicating no difference between laparoscopic and open pull-through [19–21]. At present, studies focus on the head-to-head comparison between two of open Duhamel (OD), laparoscopic-assisted Duhamel (LD), TEPT, and laparoscopic-assisted endorectal pull-through (LEPT) operations [21–24]. However, it is uncertain which of the four operations is superior in Hirschsprung disease.

To fill the above research gap, the objective of this study was to comprehensively compare and rank the effects of OD, LD, TEPT, and LEPT on operation condition and complications in Hirschsprung disease with direct and indirect evidence through a Bayesian network meta-analysis. This work may help clinicians make better surgical decisions among these four procedures when treating patients with Hirschsprung disease, thereby providing more favorable postoperative outcomes for the patients. To be noted, compared with a traditional frequentist network meta-analysis, a Bayesian approach has the following advantages: (1) it can not only effectively integrate data and flexibly build models, but also use the obtained posterior probability to rank all interventions participating in the comparison and distinguish comparative advantages and disadvantages, while a frequentist method can only rely on the effect size and its 95% confidence interval (CI) obtained by pairwise comparison in ranking; (2) since a frequentist approach uses the maximum likelihood method in parameter estimation, which estimates the maximum likelihood function through continuous iteration, it is prone to instability and biased results, while a Bayesian approach does not have this problem, so its estimated values are more accurate than those of a frequentist approach [25].

## Methods

### Search strategy

Two independent authors (XT Zhang and QQ Xu) conducted comprehensive search for the following databases: PubMed, Embase, Cochrane Library, Web of Science, CNKI, WanFang, and VIP. Disagreements were settled by another author (DM Wang). The last search time was August 4, 2022. English search terms included: “Hirschsprung Disease” OR “HSCR” OR “Hirschsprung’s Disease” OR “Disease, Hirschsprung” OR “Megacolon, Congenital” OR “Disease, Hirschsprung’s” OR “Hirschsprungs Disease” OR “Megacolon, Aganglionic” OR “Aganglionic Megacolon” OR “Congenital Megacolon” OR “Rectosigmoid Aganglionosis” OR “Aganglionosis, Rectosigmoid” OR “Congenital Intestinal Aganglionosis” OR “Aganglionosis, Colonic” OR “Colonic Aganglionosis” AND “Soave” OR “Duhamel” OR “Endorectal pull-through” OR “Transanal pull-through” OR “Transanal endorectal pull-through” OR “TEPT” OR “TERPT” OR “Laparoscopy” OR “Laparosc*” OR “Georgeson” OR “Laparoscopy-assisted pull-through” OR “LPT”.

### Inclusion and exclusion criteria

Inclusion criteria were: (1) studies on patients with Hirschsprung Disease; (2) studies on patients undergoing TEPT, LEPT, OD, and LD; (3) studies on any one or more of the following outcomes: operation-related indicators and complications; (4) randomized controlled trials (RCTs) and cohort studies.

Exclusion criteria were: (1) animal experiments; (2) studies with unclear grouping or groups of mixed surgical approaches, such as groups that did not indicate whether it was transabdominal, transanal or laparoscopic, or groups receiving open and laparoscopic treatment at the same time; (3) studies with a sample size < 5 in any group; (4) studies with incomplete data or for which data could not be extracted; (5) case reports, conference abstracts, editorial materials, protocols, theses, reviews, or meta-analyses; (6) non-English and non-Chinese studies.

### Outcomes

The outcomes were operation-related indicators and complication-related indicators. Operation-related indicators included intraoperative blood loss (mL), operating time (min), gastrointestinal function recovery time (h), and hospital stay. Complication-related indicators included incidence of complications, anastomotic stricture, anastomotic leakage, infection, intestinal obstruction, soiling, constipation, and Hirschprung-associated enterocolitis (HAEC).

### Data extraction and quality assessment

Data on first author, year of publication, country, study design, group, sample size, sex (male/female), age at surgery (months), aganglionic segment, follow-up time (months), quality assessment, and outcome were extracted by two authors (Y Li and QY Zhang) independently. To assess the quality of RCTs, the modified Jadad scale [26] was applied, which had a total score of 7 points, with 1–3 points as low quality and 4–7 points as high quality. For the quality evaluation of cohort studies, we used the modified Newcastle–Ottawa scale (NOS) [27]. The scale had a total score of 9, with 0–3 as poor quality, 4–6 as fair quality, and 7–9 as good quality. The Grading of Recommendations Assessment, Development and Evaluation (GRADE) approach [28] was used with GRADE pro GDT software to evaluate the quality of evidence in this network meta-analysis from five domains: risk of bias, inconsistency, indirectness, imprecision, and other considerations. The quality of evidence was classified into high, moderate, low, and very low.

### Statistical analysis

The Gemtc 1.0.1 package in Stata15.1 (Stata Corporation, College Station, TX, USA) and R 4.1.3 (R Foundation for Statistical Computing, Vienna, Austria) software was used for statistical analysis. The network meta-analysis was carried out by building a Bayesian framework and a Monte Carlo Markov Chain (MCMC) model. The number of model chains was 4, the number of initial iterations was 20,000, the number of updated iterations was 50,000, and the step size was 1. The I^2^ statistic was the main indicator of statistical heterogeneity, and I^2^ < 25%, 25–50% and > 50% indicated low, moderate and high heterogeneity, respectively. Consistency referred to the statistical consistency between direct and indirect effect sizes for the same comparison. The deviation information criterions (DICs) of consistency and the non-consistency models were compared, and a small value indicated a better fit. The absolute value of the difference in the DICs within 5 denoted consistency between indirect and direct evidence. For measurement data, weighted mean differences (WMDs) and 95% credibility intervals (CrIs) were reported; for enumeration data, relative risks (RRs) and 95%CrIs were calculated. Network plots, forest plots, league tables and rank probabilities were drawn for all outcomes.

## Results

### Characteristics of the included studies

**Fig. 1:**
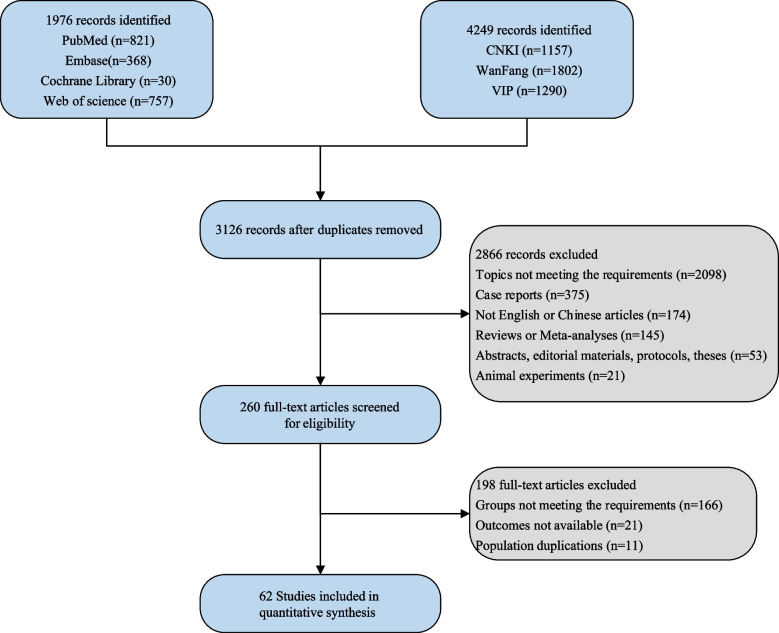
Flow chart of study selection

After comprehensive search, 1976 studies were identified from PubMed, Embase, Cochrane Library and Web of Science, and 4249 studies were retrieved from CNKI, WanFang and VIP. There were 3126 studies following de-duplication. Finally, 62 studies [14, 15, 21–24, 26–81] were included for analysis based on the inclusion and exclusion criteria. Figure 1 shows the flow chart of study selection. Of these included studies, 58 were double-arm studies, and four were three arm studies. There were 2039 TEPT patients, 1669 LEPT patients, 951 OD patients and 122 LD patients. Thirty-five articles reported the type of aganglionic segments (long, short, common, etc.); 10 reported specific aganglionic sites (rectosigmoid, descending colon, transverse colon, etc.); 17 did not mention the clinical classification of Hirschsprung disease. The included studies were published between 2005 and 2022. The baseline characteristics of the included studies are illustrated in Supplementary Table 1. Of 45 cohort studies, three had low quality, 37 had fair quality, and five had high quality. Among 17 RCTs, 16 had low quality and one had high quality.

### Operation-related indicators

#### Intraoperative blood loss

**Fig. 2:**
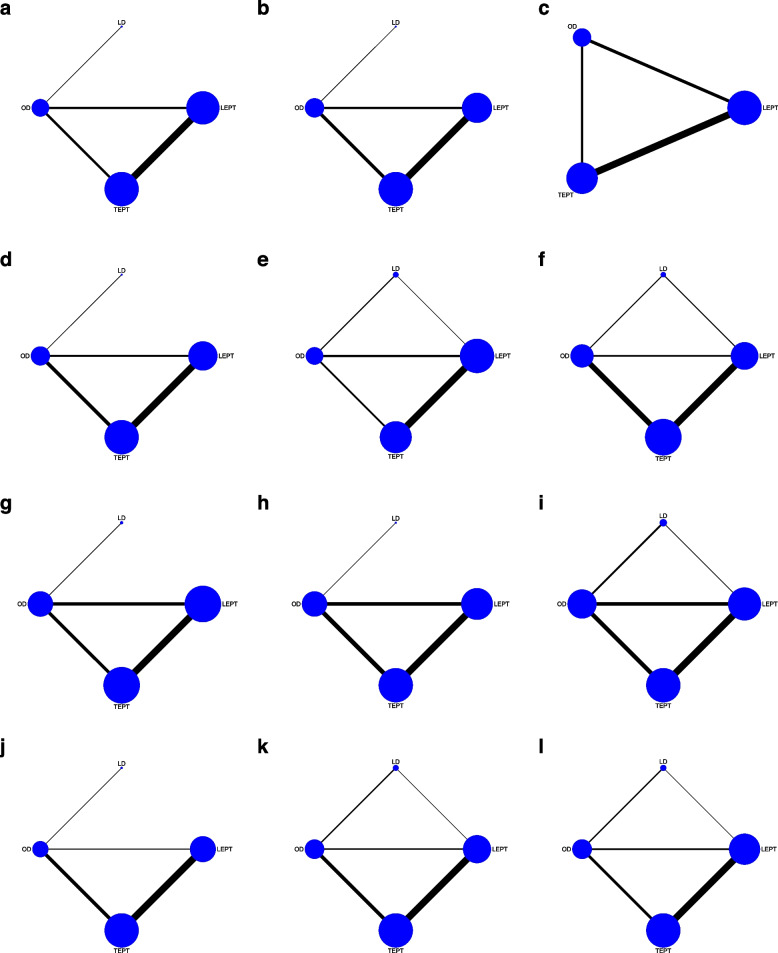
**a**-**l** Network plots of different operations for various outcomes. **a** Intraoperative blood loss; **b** operating time; **c** gastrointestinal function recovery time; **d** hospital stay; **e** incidence of complications; **f** anastomotic stricture; **g** anastomotic leakage; **h** infection; **i** intestinal obstruction; **j** soiling; **k** constipation; **l** HAEC. OD, open Duhamel; LD, laparoscopic-assisted Duhamel; TEPT, transanal endorectal pull-through; LEPT, laparoscopic-assisted endorectal pull-through; HAEC, Hirschprung-associated enterocolitis

**Fig. 3:**
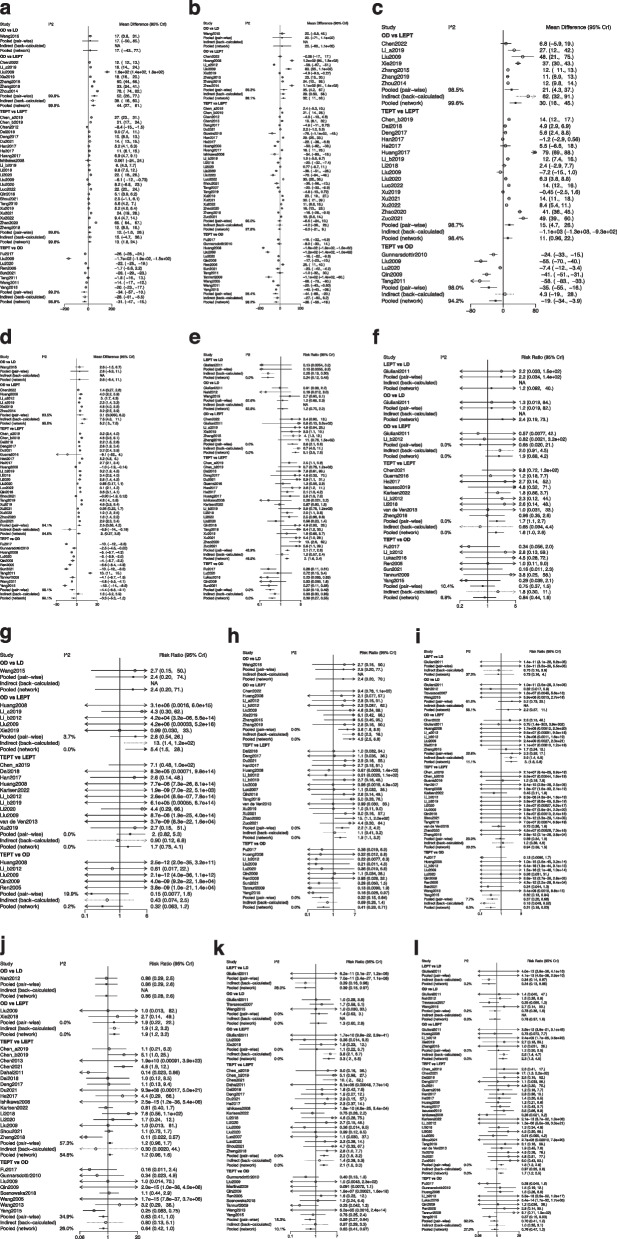
**a**-**l** Forest plots of different operations for various outcomes. **a** Intraoperative blood loss; **b** operating time; **c** gastrointestinal function recovery time; **d** hospital stay; **e** incidence of complications; **f** anastomotic stricture; **g** anastomotic leakage; **h** infection; **i** intestinal obstruction; **j** soiling; **k** constipation; **l** HAEC. OD, open Duhamel; LD, laparoscopic-assisted Duhamel; TEPT, transanal endorectal pull-through; LEPT, laparoscopic-assisted endorectal pull-through; HAEC, Hirschprung-associated enterocolitis; CrIs, credibility intervals

A total of 38 studies with 3046 patients assessed intraoperative blood loss, and OD, LD, TEPT, and LEPT were involved in network plot formation (Fig. 2a). OD was related to significantly more intraoperative blood loss than LEPT, according to the forest plot (pooled WMD = 52.00, 95%CrI: 26.00, 77.00) (Fig. 3a). The league table demonstrated that intraoperative blood loss in the OD group was more than that in the LEPT group (pooled WMD = 44.00, 95%CrI: 27.33, 60.94). Patients lost more blood during TEPT versus LEPT (pooled WMD = 13.08, 95%CrI: 1.80, 24.30) (Table 1). In terms of intraoperative blood loss, LEPT was most likely to be the optimal procedure (79.76%) (Table 2).

**Table 1:** League tables of different operations for various outcomes

Operation-related indicators				
Intraoperative blood loss				
	LD	LEPT	OD	TEPT
LD	LD	-26.75 (-89.51, 35.25)	17.23 (-42.93, 77.07)	-13.71 (-76.36, 48.18)
LEPT	26.75 (-35.25, 89.51)	LEPT	44.00 (27.33, 60.94)	13.08 (1.80, 24.30)
OD	-17.23 (-77.07, 42.93)	-44.00 (-60.94, -27.33)	OD	-30.95 (-47.55, -14.58)
TEPT	13.71 (-48.18, 76.36)	-13.08 (-24.3, -1.8)	30.95 (14.58, 47.55)	TEPT
Operating time				
	LD	LEPT	OD	TEPT
LD	LD	-12.46 (-100.47, 75.78)	19.69 (-65.89, 105.26)	-18.52 (-106.13, 69.29)
LEPT	12.46 (-75.78, 100.47)	LEPT	32.08 (11.12, 53.16)	-6.02 (-21, 8.83)
OD	-19.69 (-105.26, 65.89)	-32.08 (-53.16, -11.12)	OD	-38.11 (-57.84, -18.55)
TEPT	18.52 (-69.29, 106.13)	6.02 (-8.83, 21)	38.11 (18.55, 57.84)	TEPT
Gastrointestinal function recovery time				
	LEPT	OD	TEPT	
LEPT	LEPT	30.39 (16.08, 44.94)	11.49 (0.96, 22.05)	
OD	-30.39 (-44.94, -16.08)	OD	-18.88 (-34.18, -3.94)	
TEPT	-11.49 (-22.05, -0.93)	18.88 (3.94, 34.18)	TEPT	
Hospital stay				
	LD	LEPT	OD	TEPT
LD	LD	-2.64 (-11.82, 6.52)	2.59 (-6.36, 11.45)	-0.65 (-9.81, 8.45)
LEPT	2.64 (-6.52, 11.82)	LEPT	5.24 (2.98, 7.47)	1.99 (0.37, 3.58)
OD	-2.59 (-11.45, 6.36)	-5.24 (-7.47, -2.98)	OD	-3.25 (-5.32, -1.17)
TEPT	0.65 (-8.45, 9.81)	-1.99 (-3.58, -0.37)	3.25 (1.17, 5.32)	TEPT
Complication-related indicators				
Incidence of complications				
	LD	LEPT	OD	TEPT
LD	LD	0.24 (0.12, 0.48)	1.24 (0.7, 2.19)	0.49 (0.25, 0.95)
LEPT	4.05 (2.07, 8.01)	LEPT	5.10 (3.48, 7.45)	1.98 (1.63, 2.42)
OD	0.81 (0.46, 1.42)	0.2 (0.13, 0.29)	OD	0.40 (0.27, 0.55)
TEPT	2.05 (1.05, 4)	0.51 (0.41, 0.61)	2.52 (1.79, 3.68)	TEPT
Anastomotic stricture				
	LD	LEPT	OD	TEPT
LD	LD	1.26 (0.09, 39.34)	2.40 (0.20, 73.68)	2.02 (0.16, 63.58)
LEPT	0.8 (0.03, 10.55)	LEPT	1.91 (0.89, 4.22)	1.61 (1.03, 2.56)
OD	0.42 (0.01, 5.09)	0.52 (0.24, 1.13)	OD	0.84 (0.44, 1.62)
TEPT	0.49 (0.02, 6.41)	0.62 (0.39, 0.97)	1.19 (0.62, 2.29)	TEPT
Anastomotic leakage				
	LD	LEPT	OD	TEPT
LD	LD	0.44 (0.02, 16.43)	2.38 (0.2, 69.04)	0.77 (0.04, 28.24)
LEPT	2.26 (0.06, 42.2)	LEPT	5.35 (1.45, 27.68)	1.72 (0.75, 4.12)
OD	0.42 (0.01, 5.05)	0.19 (0.04, 0.69)	OD	0.32 (0.07, 1.24)
TEPT	1.31 (0.04, 24.62)	0.58 (0.24, 1.33)	3.09 (0.81, 15.02)	TEPT
Infection				
	LD	LEPT	OD	TEPT
LD	LD	0.54 (0.04, 16.42)	2.45 (0.21, 70.47)	1.01 (0.08, 30.29)
LEPT	1.85 (0.06, 23.91)	LEPT	4.52 (2.45, 8.84)	1.87 (1.13, 3.18)
OD	0.41 (0.01, 4.85)	0.22 (0.11, 0.41)	OD	0.41 (0.23, 0.71)
TEPT	0.99 (0.03, 12.55)	0.53 (0.31, 0.89)	2.42 (1.41, 4.29)	TEPT
Intestinal obstruction				
	LD	LEPT	OD	TEPT
LD	LD	0.72 (0.16, 3.91)	2.16 (0.57, 10.58)	0.68 (0.16, 3.57)
LEPT	1.38 (0.26, 6.08)	LEPT	3.00 (1.60, 5.79)	0.94 (0.59, 1.49)
OD	0.46 (0.09, 1.75)	0.33 (0.17, 0.63)	OD	0.31 (0.18, 0.53)
TEPT	1.47 (0.28, 6.22)	1.06 (0.67, 1.69)	3.19 (1.9, 5.5)	TEPT
Soiling				
	LD	LEPT	OD	TEPT
LD	LD	0.45 (0.13, 1.49)	0.86 (0.28, 2.59)	0.55 (0.17, 1.81)
LEPT	2.23 (0.67, 7.51)	LEPT	1.91 (1.16, 3.17)	1.23 (0.96, 1.64)
OD	1.17 (0.39, 3.52)	0.52 (0.32, 0.86)	OD	0.64 (0.42, 0.99)
TEPT	1.81 (0.55, 5.88)	0.81 (0.61, 1.04)	1.55 (1, 2.38)	TEPT
Constipation				
	LD	LEPT	OD	TEPT
LD	LD	0.39 (0.15, 0.97)	1.29 (0.6, 2.78)	0.81 (0.34, 1.94)
LEPT	2.57 (1.03, 6.48)	LEPT	3.31 (1.95, 5.74)	2.09 (1.47, 3.04)
OD	0.78 (0.36, 1.67)	0.3 (0.17, 0.51)	OD	0.63 (0.41, 0.97)
TEPT	1.23 (0.51, 2.93)	0.48 (0.33, 0.68)	1.58 (1.03, 2.45)	TEPT
HAEC				
	LD	LEPT	OD	TEPT
LD	LD	0.34 (0.13, 0.85)	0.78 (0.37, 1.62)	0.59 (0.24, 1.41)
LEPT	2.95 (1.17, 7.55)	LEPT	2.29 (1.31, 4.04)	1.74 (1.24, 2.45)
OD	1.28 (0.62, 2.74)	0.44 (0.25, 0.76)	OD	0.76 (0.47, 1.22)
TEPT	1.7 (0.71, 4.12)	0.58 (0.41, 0.81)	1.32 (0.82, 2.13)	TEPT

The values in the table were WMDs/RRs and 95%CrIs

*OD* open Duhamel, *LD* laparoscopic-assisted Duhamel, *TEPT* transanal endorectal pull-through, *LEPT* laparoscopic-assisted endorectal pull-through, *HAEC* Hirschsprung-associated enterocolitis, *WMD* weighted mean difference, *RR* relative risk, *CrI* credibility interval

**Table 2:** Rank probabilities of different operations for various outcomes

Operation-related indicators				
Intraoperative blood loss				
	[1]	[2]	[3]	[4]
LD	0.281045	0.3906	0.13497	0.193385
LEPT	0	0.00347	0.1989	0.79763
OD	0.71879	0.28115	0.00006	0
TEPT	0.000165	0.32478	0.66607	0.008985
Operating time				
	[1]	[2]	[3]	[4]
LD	0.32367	0.27992	0.06709	0.32932
LEPT	0.001075	0.31881	0.539815	0.1403
OD	0.675185	0.32413	0.000655	0.00003
TEPT	0.00007	0.07714	0.39244	0.53035
Gastrointestinal function recovery time				
	[1]	[2]	[3]	
LEPT	0.000035	0.01714	0.982825	
OD	0.99229	0.007645	0.000065	
TEPT	0.007675	0.975215	0.01711	
Hospital stay				
	[1]	[2]	[3]	[4]
LD	0.280445	0.276005	0.162235	0.281315
LEPT	0.000005	0.003705	0.283235	0.713055
OD	0.718435	0.28098	0.000585	0
TEPT	0.001115	0.43931	0.553945	0.00563
Complication-related indicators				
Incidence of complications				
	[1]	[2]	[3]	[4]
LD	0.228725	0.75379	0.01746	0.000025
LEPT	0	0	0.000025	0.999975
OD	0.771275	0.228725	0	0
TEPT	0	0.017485	0.982515	0
Anastomotic stricture				
	[1]	[2]	[3]	[4]
LD	0.23155	0.07736	0.12663	0.56446
LEPT	0.00458	0.041005	0.538455	0.41596
OD	0.531655	0.354305	0.099425	0.014615
TEPT	0.232215	0.52733	0.23549	0.004965
Anastomotic leakage				
	[1]	[2]	[3]	[4]
LD	0.244165	0.32354	0.135745	0.29655
LEPT	0.001175	0.03869	0.324485	0.63565
OD	0.71334	0.27057	0.015245	0.000845
TEPT	0.04132	0.3672	0.524525	0.066955
Infection				
	[1]	[2]	[3]	[4]
LD	0.237835	0.258085	0.17163	0.33245
LEPT	0	0.003275	0.333515	0.66321
OD	0.761655	0.238175	0.00017	0
TEPT	0.00051	0.500465	0.494685	0.00434
Intestinal obstruction				
	[1]	[2]	[3]	[4]
LD	0.130675	0.500645	0.087535	0.281145
LEPT	0.00021	0.23654	0.467995	0.295255
OD	0.869115	0.13086	0.000025	0
TEPT	0	0.131955	0.444445	0.4236
Soiling				
	[1]	[2]	[3]	[4]
LD	0.611875	0.23028	0.06715	0.090695
LEPT	0.000795	0.01029	0.12732	0.861595
OD	0.37676	0.606	0.014445	0.002795
TEPT	0.01057	0.15343	0.791085	0.044915
Constipation				
	[1]	[2]	[3]	[4]
LD	0.25258	0.42798	0.297565	0.021875
LEPT	0	0.000005	0.02188	0.978115
OD	0.733585	0.26147	0.004945	0
TEPT	0.013835	0.310545	0.67561	0.00001
HAEC				
	[1]	[2]	[3]	[4]
LD	0.734925	0.162675	0.09197	0.01043
LEPT	0.00002	0.000815	0.011575	0.98759
OD	0.21855	0.686325	0.093725	0.0014
TEPT	0.046505	0.150185	0.80273	0.00058

*OD* open Duhamel, *LD* laparoscopic-assisted Duhamel, *TEPT* transanal endorectal pull-through, *LEPT* laparoscopic-assisted endorectal pull-through, *HAEC* Hirschsprung-associated enterocolitis

#### Operating time

Data on operating time were provided by 45 studies on 3499 patients. OD, LD, TEPT, and LEPT were compared (Fig. 2b). The forest plot illustrated that compared with patients undergoing LEPT, those undergoing OD had significantly longer operating time (pooled WMD = 35.00, 95%CrI: 4.20, 67.00). Operating time in the TEPT group was significantly shorter than that in the OD group (pooled WMD = -44.00, 95%CrI: -69.00, -20.00) (Fig. 3b). According to the league table, the OD group had a significantly longer operating time than the LEPT group (pooled WMD = 32.08, 95%CrI: 11.12, 53.16). TEPT was associated with significantly decreased operating time in contrast to OD (pooled WMD = -38.11, 95%CrI: -57.84, -18.55) (Table 1). Patients with TEPT had the greatest possibility to have the shortest operating time (53.04%), as presented by the rank probability (Table 2).

#### Gastrointestinal function recovery time

Twenty-six studies with 1887 patients assessed OD, TEPT and LEPT for gastrointestinal function recovery time (Fig. 2c). In view of the forest plot, the OD group was found to have significantly longer gastrointestinal function recovery time than the LEPT group (pooled WMD = 21.00, 95%CrI: 4.30, 37.00). Gastrointestinal function recovery time in the TEPT group was significantly longer than that in the LEPT group (pooled WMD = 15.00, 95%CrI: 4.70, 26.00) (Fig. 3c). Based on the league table, patients undergoing OD had significantly longer gastrointestinal function recovery time, as compared with those undergoing LEPT (pooled WMD = 30.39, 95%CrI: 16.08, 44.94). The TEPT group had significantly longer gastrointestinal function recovery time than the LEPT group (pooled WMD = 11.49, 95%CrI: 0.96, 22.05) (Table 1). The rank probability showed that LEPT was most likely to be the best operation regarding gastrointestinal function recovery time (98.28%) (Table 2).

#### Hospital stay

Hospital stay was evaluated in 38 studies with 2861 patients. There were comparisons among OD, LD, TEPT, and LEPT (Fig. 2d). Based on the forest plot, the OD group had significantly longer hospital stay than the LEPT group (pooled WMD = 3.10, 95%CrI: 0.005, 6.20). TEPT was associated with significantly prolonged hospital stay versus LEPT (pooled WMD = 2.50, 95%CrI: 0.86, 4.20) (Fig. 3d). As exhibited by the league table, longer hospital stay was observed in patients with OD versus LEPT (pooled WMD = 5.24, 95%CrI: 2.98, 7.47). Hospital stay in the TEPT group was significantly longer than that in the LEPT group (pooled WMD = 1.99, 95%CrI: 0.37, 3.58) (Table 1). With the rank probability, LEPT had the highest possibility to be the most effective operation with respect to hospital stay (71.31%) (Table 2).

### Complication-related indicators

#### Incidence of complications

Thirty-four studies with 2550 patients investigated OD, LD, TEPT, and LEPT for the incidence of complications (Fig. 2e). The forest plot showed that the OD group had a significantly higher incidence of complications than the LEPT group (pooled RR = 3.60, 95%CrI: 2.10, 6.60). The incidence of complications in the TEPT group was significantly greater than that in the LEPT group (pooled RR = 2.10, 95%CrI: 1.70, 2.60) (Fig. 3e). From the league table, the significantly reduced incidence of complications was found in the LEPT group versus the LD group (pooled RR = 0.24, 95%CrI: 0.12, 0.48). Compared with LEPT, OD was associated with a significantly increased incidence of complications (pooled RR = 5.10, 95%CrI: 3.48, 7.45). Patients undergoing TEPT had a significantly greater incidence of complications than those undergoing LEPT (pooled RR = 1.98, 95%CrI: 1.63, 2.42) (Table 1). The rank probability indicated that for complications, LEPT is most likely to have the best effect (99.99%) (Table 2).

#### Anastomotic stricture

Concerning anastomotic stricture, LEPT, TEPT, OD, and LD were assessed with 16 studies of 1594 patients (Fig. 2f). As exhibited by the forest plot, the TEPT group had a significantly elevated incidence of anastomotic stricture in contrast to the LEPT group (pooled RR = 1.70, 95%CrI: 1.10, 2.70) (Fig. 3f). The league table demonstrated that the incidence of anastomotic stricture in patients undergoing TEPT was significantly higher than that in those undergoing LEPT (pooled RR = 1.61, 95%CrI: 1.03, 2.56) (Table 1). According to the rank probability, LD was most likely to be the optimum operation with respect to anastomotic stricture (56.45%) (Table 2).

#### Anastomotic leakage

Anastomotic leakage was estimated in 16 studies on 1313 patients which involved LEPT, TEPT, OD, and LD (Fig. 2g). Based on the forest plot, no significant difference was found in the incidence of anastomotic leakage between OD and LD, between OD and LEPT, between TEPT and LEPT, and between TEPT and OD (Fig. 3g). The league table illustrated that compared with the LEPT group, the OD group had a significantly increased incidence of anastomotic leakage (pooled RR = 5.35, 95%CrI: 1.45, 27.68) (Table 1). The rank probability showed that LEPT had the highest likelihood to be the best operation regarding anastomotic leakage (63.57%) (Table 2).

#### Infection

Twenty-nine studies of 2444 patients reported infection after LEPT, TEPT, OD, and LD (Fig. 2h). Based on the forest plot, the OD group had a significantly higher incidence of infection than the LEPT group (pooled RR = 3.60, 95%CrI: 1.60, 8.90). The TEPT group had a significantly greater incidence of infection than the LEPT group (pooled RR = 2.20, 95%CrI: 1.20, 4.20) (Fig. 3h). The league table showed that the incidence of infection in the OD group was significantly higher than that in the LEPT group (pooled RR = 4.52, 95%CrI: 2.45, 8.84). The TEPT group had a significantly increased incidence of infection than the LEPT group (pooled RR = 1.87, 95%CrI: 1.13, 3.18) (Table 1). The rank probability indicated that LEPT is most likely to be the best operation concerning infection (66.32%) (Table 2).

#### Intestinal obstruction

LEPT, TEPT, OD, and LD were compared with 31 studies on 2612 patients for intestinal obstruction (Fig. 2i). The forest plot showed that compared with OD, TEPT was associated with a significantly reduced incidence of intestinal obstruction (pooled RR = 0.37, 95%CrI: 0.20, 0.68) (Fig. 3i). The OD group had a significantly higher incidence of intestinal obstruction than the LEPT group (pooled RR = 3.00, 95%CrI: 1.60, 5.79). The incidence of intestinal obstruction was significantly lower in the TEPT group versus the OD group (pooled RR = 0.31, 95%CrI: 0.18, 0.53), as shown in the league table (Table 1). The rank probability exhibited that TEPT had the greatest probability not to develop intestinal obstruction (42.36%) (Table 2).

#### Soiling

Twenty-five studies with 1903 patients assessed soiling, involving LEPT, TEPT, OD, and LD (Fig. 2j). According to the forest plot, no significant differences were found in soiling between OD and LD, between OD and LEPT, between TEPT and LEPT, and between TEPT and OD (Fig. 3j). The league table demonstrated that compared with LEPT, OD was associated with a significantly higher incidence of soiling (pooled RR = 1.91, 95%CrI: 1.16, 3.17) (Table 1). Based on the rank probability, patients with LEPT had the greatest likelihood not to develop soiling (86.16%) (Table 2).

#### Constipation

For constipation, 30 studies with 2148 patients were included to depict the network plot for OD, LD, TEPT and LEPT (Fig. 2k). The forest plot illustrated that patients with TEPT had a significantly higher incidence of constipation than those with LEPT (pooled RR = 2.20, 95%CrI: 1.50, 3.20) (Fig. 3k). The league table showed that in contrast to LD, LEPT was significantly more effective in reducing the incidence of constipation (pooled RR = 0.39, 95%CrI: 0.15, 0.97) (Table 1). As demonstrated by the rank probability, LEPT was most likely not to result in constipation (97.81%) (Table 2).

#### HAEC

HAEC was evaluated in 36 studies of 3041 patients, and OD, LD, TEPT and LEPT were compared (Fig. 2l). The forest plot exhibited that patients with TEPT had a significantly higher incidence of HAEC than those with LEPT (pooled RR = 1.80, 95%CrI: 1.30, 2.60) (Fig. 3l). According to the league table, LEPT was associated with a significantly lower incidence of HAEC than LD (pooled RR = 0.34, 95%CrI: 0.13, 0.85). The OD group had a significantly higher incidence of HAEC than the LEPT group (pooled RR = 2.29, 95%CrI: 1.31, 4.0). The incidence of HAEC was significantly greater in the TEPT group versus the LEPT group (pooled RR = 1.74, 95%CrI: 1.24, 2.45) (Table 1). As demonstrated by the rank probability, LEPT was most likely to be the optimal operation in terms of HAEC (98.76%) (Table 2).

## Discussion

To the best of our knowledge, this network meta-analysis comprehensively evaluated, compared and ranked the efficacy of OD, LD, TEPT and LEPT to investigate the optimal surgical method in Hirschsprung disease for the first time. The results demonstrated that LEPT may be the optimal operation in improving operation condition and complications, compared with OD, LD and TEPT, which might serve as a reference for clinical decision-making in treating Hirschsprung disease.

At present, the therapeutic effects of two of the four operations have been compared in meta-analyses. Mao et al. [6] compared Duhamel and TEPT operations in Hirschsprung disease via combined analysis of six studies with 280 patients, and found that children treated with the two interventions had similar rates of postoperative fecal incontinence and operation time, while Duhamel operation was related to longer postoperative hospital stay and a lower rate of enterocolitis. The systematic review and meta-analysis of Scholfield et al. [82] compared long-term outcomes for OD and LD procedures in Hirschsprung disease with 11 studies of 456 patients, and showed the advantage of LD over OD as regards incidences of soiling/incontinence and further surgery, hospital stay, time to oral feed, although OD had shorter operation time. In the analysis of Seo et al. [5] comparing Duhamel and TEPT based on seven studies of 430 patients, patients undergoing Duhamel operation appeared to have a lower incidence of anastomotic stricture, and the incidences of postoperative incontinence/soiling and anastomotic leakage were comparable in the two groups. Yan et al. [83] evaluated the clinical outcomes of TEPT and transabdominal surgery (including the Duhamel procedure) with 10 studies of 724 patients, and demonstrated that TEPT was better than transabdominal approach concerning hospital stay, postoperative incontinence and constipation. Zhang et al. [17] compared laparoscopic-assisted and laparotomy approaches of procedures including Duhamel and Soave by pooling 16 studies with 774 patients, and found that patients with laparoscopic-assisted operations had lower estimated blood loss, hospital stay, mean first bowel movement, and number of complications. In another meta-analysis of 9 articles with 421 patients comparing laparoscopic-assisted surgery and open surgery (involving OD, LD, TEPT and LEPT), the laparoscopic-assisted surgery group exhibited less operation time, intraoperative blood loss and postoperative hospital stay, and fewer complications [84]. Further, the current network meta-analysis compared OD, LD, TEPT and LEPT using direct and indirect evidence from 62 studies of 4781 patients, and illustrated that as regards operation-related indicators, patients undergoing LEPT may have least intraoperative blood loss, minimum gastrointestinal function recovery time, and shortest hospital stay; for complication-related indicators, LEPT may be the optimal procedure in terms of complications, anastomotic leakage, infection, soiling, constipation, and HAEC.

Concerning operation-related indicators, LEPT and TEPT may have advantages over OD and LD in terms of operating time, intraoperative blood loss, gastrointestinal function recovery time, and hospital stay. This may be attributed to that fewer steps are involved in TEPT and LEPT operations, and most of the steps are completed through the anus, resulting in less anatomical dissociation of the pelvic cavity and less overall damage [8]. On the contrary, OD and LD require relatively extensive dissociation of the pelvic cavity to complete side-to-side anastomosis of the proximal colon and distal rectum [85], which may cause greater overall damage. Moreover, OD also requires abdominal surgery, which can cause more trauma than LD. The peristalsis, texture, thickness and color of the colon in patients can be observed intuitively through the magnifying effect of laparoscopy during LEPT, so as to judge the resection plane in time, and determine the lesion site to be removed according to intraoperative freezing results. Besides, the dissociation of the pelvic floor structure and the anatomy of the rectum is more accurate with LEPT, allowing for accurate observation of the blood flow of the pulled-out bowel, thereby removing all lesions at once [86]. Compared with LEPT, the operation of colon dissociation in TEPT is often based on the operator’s experience, and the lack of intuitive comparison during the dissociation process can easily lead to inappropriate and imprecise operation, which may cause defects in TEPT in many aspects. Given the above possible reason, as a minimally invasive operation, LEPT with the help of laparoscopy may reduce the intraoperative injury of patients, reduce blood loss, and promote early recovery. As for operating time, TEPT exhibited the highest likelihood to be the best procedure, despite no significant difference between LEPT and TEPT. A potential explanation may be that TEPT does not need to set up a laparoscope for intra-abdominal free operations, and the relative operation time is shorter.

With respect to complication-related indicators, patients undergoing LEPT may have the lowest incidences of complications, anastomotic leakage, infection, soiling, constipation, and HAEC. With LEPT, the diseased bowel is not removed in the abdominal cavity and the exposed area is small. Additionally, accurate intraoperative operation of LEPT may fully ensure that there is no tension at the anal anastomotic stoma. These advantages may reduce many early complications, including infection and anastomotic leakage [87, 88]. As for soiling, compared with OD and LD, LEPT does not require laparotomy, and has the advantages of reducing the chance of surgical trauma and abdominal cavity pollution, not separating the perirectal area during the operation, less pelvic nerve injury, retaining the internal and external sphincters, rapid recovery, and low incidence of soiling. In TEPT operation, the occurrence of soiling is mostly related to excessive pulling of the anus during operation [89], while laparoscopy-assisted approach can effectively avoid excessive pulling, fully free the colonic ligament and mesentery, which is conducive to the retention of the colonic stool storage function, thereby reducing the risk of soiling. In regard to constipation, TEPT, on the one hand, only peels off the rectal mucosa, but does not remove the rectal muscle sheath. The lack of ganglion cells in the rectal muscle sheath after the surgery may lead to constipation, affecting the prognosis of patients. On the other hand, for the scope of muscle sheath preservation, the shorter the rectal muscle sheath preservation, the lower the incidence of postoperative enteritis and constipation. LEPT may reduce the incidence of postoperative constipation by partially removing the muscle sheath of the posterior rectal wall in a strip or wedge shape through laparoscopy [41]. Besides, the Duhamel procedure, including LD and OD, is easily associated with constipation because of the retained aganglionic rectal pouch [90]. Regarding anastomotic stricture, LD may be the superior operation. This may be attributed to that the anastomotic stoma under the Duhamel procedure is relatively large and is not prone to stenosis; compared with OD, the anatomical process of LD is performed under a laparoscope, which is clearer, and the anastomosis is relatively more accurate. Patients undergoing TEPT and LEPT are prone to secondary intrathecal infection and then anastomotic stenosis. Since LEPT is performed under direct vision, the incidence of anastomotic stricture may be lower in LEPT than in TEPT. Regarding intestinal obstruction, blind pouch and gate syndromes are specific complications under the Duhamel operation. When the anal sphincter contracts, feces are pressed forward into the blind pouch, which can form fecal stones over time, compressing the posterior colon, and causing mechanical obstruction. However, both TEPT and LEPT can effectively avoid blind pouch and gate syndromes. Moreover, compared with TEPT and LEPT, the degree of abdominal cavity dissociation in the Duhamel operation is relatively large, which may easily result in secondary adhesive intestinal obstruction [91]. The remaining segments of aganglionosis and dysbacteriosis due to anastomotic stricture and obstruction may be related to HACE [92]. Compared with TEPT, LEPT could remove aganglionic sheaths as much as possible, and meanwhile, LEPT could also have a low rate about anastomotic stricture and intestinal obstruction. These factors may result in LEPT not being prone to HACE. For the increased incidence of complications (including infective) with TEPT than LEPT, possible explanations are as follows: the LEPT procedure is more intuitive, avoiding uncertainty during the process of transanal pull-through in TEPT. Besides, LEPT is performed more thoroughly, and both TEPT and LEPT require a certain proportion of muscle sheath to be retained. However, compared with TEPT, LEPT can achieve shorter retention of muscle sheath, which not only avoids recurrence but also to some extent avoids the risk of intrathecal infection.

Although many studies showed the advantage of laparoscopic method over the open pull-through and laparoscopic is now commonly performed even in low-middle income countries, there were still studies indicating no difference between laparoscopic and open pull-through [19–21]. Additionally, in clinical practice, compared with TEPT, LEPT faces relative disadvantages such as insufficient support for laparoscopy due to weak abdominal walls in children, longer learning cycles, and larger pelvic anatomy range, high skill requirements for operators, and longer surgical time. Therefore, some clinicians tend to choose TEPT. Given the consistent comparison results between TEPT and LEPT, and inconclusive results of the optimal surgical procedure in OD, LD, TEPT, and LEPT, this network meta-analysis was necessary to comprehensively compare and rank the effects of OD, LD, TEPT, and LEPT on operation condition and complications in Hirschsprung disease. Based on our findings from pooled analysis of 62 studies, physicians could choose LEPT in the treatment of patients with Hirschsprung disease, combined with their clinical experience and patient preference, to facilitate the recovery of patients, with a lower incidence of complications. This study can further provide credibility for the priority of LEPT clinical application, further providing theoretical basis for the clinical promotion of LEPT. Considering the use of laparoscopic instrumentation in LEPT, some measures should be taken to reduce the medical burden on patients. For example, the government should increase investment in healthcare and formulate corresponding policies to reduce healthcare costs. The government can expand medical resources, improve medical facilities, and increase the number of medical personnel by increasing the medical budget. Besides, the establishment and improvement of relevant medical insurance systems are also important means of reducing the medical burden, providing corresponding subsidies to citizens, and ensuring that low-income group can afford insurance costs and medical expenses. Some limitations should be mentioned. First, different surgical methods may be adopted when the disease occurred in different intestinal segments, but most of the included studies did not distinguish the surgical methods according to the pathological location, and subgroup analysis could not be conducted. Second, the quality of some included studies was not high, which may affect the reliability of research evidence, and most of the included studies were observational studies, which may led to the low level of evidence for some outcomes. Third, studies in other languages were not included in this analysis.

## Conclusion

LEPT may be the superior operation to OD, LD and TEPT in improving operation condition and complications, which might serve as a therapeutic choice for Hirschsprung disease. More studies are warranted to certify our findings.

### Supplementary Information

**Supplementary Material 1.**

## Data Availability

The datasets used and/or analyzed during the current study are available from the corresponding author on reasonable request.

## Abbreviations

TEPT: Transanal endorectal pull-through
OD: Open Duhamel
LD: Laparoscopic-assisted Duhamel
LEPT: Laparoscopic-assisted endorectal pull-through
HAEC: Hirschprung-associated enterocolitis
NOS: Newcastle–Ottawa scale
GRADE: Grading of Recommendations Assessment, Development and Evaluation
DICs: Deviation information criterions
WMDs: Weighted mean differences
CrIs: Credibility intervals
RRs: Relative risks
