# Supplementary material for: Duhamel and transanal endorectal pull-throughs for Hirschsprung disease: a Bayesian network meta-analysis
Source: BMC Surg. 2024 May 3;24:132. doi: 10.1186/s12893-024-02416-0 (PMC11067296; doi:10.1186/s12893-024-02416-0)
Supplement: Supplementary file 1 — Supplementary Material 1. [file 12893_2024_2416_MOESM1_ESM.docx]

**Supplementary Table 1** Baseline characteristics of the included studies.

| Author | Year | Country | Study design | Group | sample size | Sex (M/F) | Age at surgery | Aganglionic segment | Follow-up time | Quality assessment | Outcome |
| --- | --- | --- | --- | --- | --- | --- | --- | --- | --- | --- | --- |
| Chen | 2022 | China | RCT | LEPT | 32 | 18/14 | 38.9±2.21 | Short 5, common 27 | 6 | 3 | Operating time, intraoperative blood loss, gastrointestinal function recovery time, hospital stay, complications, intestinal obstruction, infection |
|  |  |  |  | OD | 32 | 20/12 | 30.3±1.13 | Long 6, short 2, common 22 |  |  |  |
| Karlsen | 2022 | Norway | Retrospective cohort | LEPT | 45 | 36/9 | 5.5 (0.6–98) | Rectosigmoid | - | 6 | Complications, HAEC, anastomotic leakage, anastomotic stricture, intestinal obstruction, soiling, constipation |
|  |  |  |  | TEPT | 46 | 37/9 | 1.9 (0.4–133) |  |  |  |  |
| Luo | 2022 | China | Cohort | LEPT | 31 | 19/12 | 7.01 ± 2.51 | Long 5, short 10, common 16 | 12 | 6 | Operating time, intraoperative blood loss, gastrointestinal function recovery time, hospital stay, constipation |
|  |  |  |  | TEPT | 31 | 17/14 | 7.52 ± 2.62 | Long 6, short 7, common 18 |  |  |  |
| Xu | 2022 | China | Retrospective cohort | LEPT | 40 | 22/18 | 13.8 ± 3.72 | Long | 6 | 6 | Operating time, intraoperative blood loss, gastrointestinal function recovery time, hospital stay |
|  |  |  |  | TEPT | 40 | 21/19 | 12.96 ± 2.88 |  |  |  |  |
| Chen | 2021 | China | Retrospective cohort | LEPT | 55 | 34/21 | 38.52±16.08 | Long 13, short 18, common 24 | ≥12 | 6 | HAEC, anastomotic stricture, soiling, constipation |
|  |  |  |  | TEPT | 55 | 31/24 | 37.56±17.04 | Long 11, short 19, common 25 |  |  |  |
| Du | 2021 | China | Cohort | LEPT | 48 | 35/13 | 34.32 ± 11.64 | - | 6 | 6 | Operating time, intraoperative blood loss, hospital stay, complications, HAEC, infection, soiling, constipation |
|  |  |  |  | TEPT | 45 | 33/12 | 32.16 ± 10.08 |  |  |  |  |
| Shou | 2021 | China | Retrospective cohort | LEPT | 15 | 10/5 | 10.3 ± 13.3 | Short 7, common 8 | - | 6 | Operating time, intraoperative blood loss, hospital stay, HAEC, intestinal obstruction, soiling, constipation |
|  |  |  |  | TEPT | 27 | 20/7 | 8.5 ± 12.0 | Short 23, common 4 |  |  |  |
| Sun | 2021 | China | RCT | TEPT | 30 | 17/13 | 15.31± 1.66 | Long 8, short 10, common 12 | 0.1 | 3 | Operating time, intraoperative blood loss, hospital stay, complications, anastomotic stricture, intestinal obstruction, infection |
|  |  |  |  | OD | 30 | 18/12 | 15.01± 1.96 | Long 7, short 12, common 11 |  |  |  |
| Xu | 2021 | China | Retrospective cohort | LEPT | 48 | 25/23 | 1.40 ± 0.81 | - | 12 | 7 | Operating time, intraoperative blood loss, gastrointestinal function recovery time, hospital stay, complications, HAEC, infection |
|  |  |  |  | TEPT | 44 | 24/20 | 1.59 ± 0.89 |  |  |  |  |
| Zuo | 2021 | China | RCT | LEPT | 43 | 24/19 | 33.00±8.76 | - | 3 | 3 | Operating time, gastrointestinal function recovery time, hospital stay, complications, HAEC, infection |
|  |  |  |  | TEPT | 43 | 22/21 | 33.24±8.64 |  |  |  |  |
| Li | 2020 | China | Cohort | LEPT | 28 | 15/13 | 12.0 ± 3.6 | Short 28 | 12 | 6 | Operating time, intraoperative blood loss, hospital stay, complications, HAEC, anastomotic leakage, intestinal obstruction, soiling, constipation |
|  |  |  |  | TEPT | 28 | 16/12 | 10.8 ± 4.8 | Long 1, short 27 |  |  |  |
| Liu | 2020 | China | Cohort | LEPT | 27 | 17/10 | 21.51±25.48 | Long 3, short 8, common 16 | 3 | 6 | Operating time, intraoperative blood loss, gastrointestinal function recovery time, hospital stay, complications, HAEC, constipation |
|  |  |  |  | TEPT | 27 | 21/6 | 25.07±25.63 | Long 2, short 12, common 13 |  |  |  |
| Lu | 2020 | China | RCT | TEPT | 34 | 14/20 | 5.58 ± 2.13 | - | 0.2 | 3 | Operating time, intraoperative blood loss, gastrointestinal function recovery time, hospital stay, complications, intestinal obstruction, infection |
|  |  |  |  | OD | 34 | 13/21 | 6.33 ± 1.05 |  |  |  |  |
| Zhao | 2020 | China | RCT | LEPT | 50 | 26/24 | 23.5 ± 1.3 | - | 1 | 3 | Operating time, intraoperative blood loss, gastrointestinal function recovery time, hospital stay, complications, intestinal obstruction, infection |
|  |  |  |  | TEPT | 50 | 27/23 | 23.6 ± 1.4 |  |  |  |  |
| Chen_a | 2019 | China | Cohort | LEPT | 57 | - | 58 (2-112) | Short 57 | 12 | 7 | Operating time, intraoperative blood loss, hospital stay, complications, HAEC, anastomotic leakage, intestinal obstruction, soiling, constipation |
|  |  |  |  | TEPT | 50 |  | 56 (3-115) | Long 2, short 48 |  |  |  |
| Chen_b | 2019 | China | RCT | LEPT | 27 | 15/12 | 37.44 ± 3.36 | - | - | 2 | Operating time, intraoperative blood loss, gastrointestinal function recovery time, hospital stay, complications, intestinal obstruction, soiling, constipation |
|  |  |  |  | TEPT | 26 | 14/12 | 36.96 ± 3.48 |  |  |  |  |
| Iacusso | 2019 | Italy | Retrospective cohort | LEPT | 46 | - | 6.3 | - | 1 | 5 | HAEC, anastomotic stricture |
|  |  |  |  | TEPT | 61 |  | 2.3 |  |  |  |  |
| Li_a | 2019 | China | RCT | LEPT | 25 | 11/14 | 44.28±6.72 | - | - | 2 | Intraoperative blood loss, gastrointestinal function recovery time, hospital stay, complications, anastomotic leakage |
|  |  |  |  | OD | 25 | 12/13 | 44.76±7.32 |  |  |  |  |
| Li_b | 2019 | China | RCT | LEPT | 15 | 10/5 | 30.0±15.6 | Long 5, short 6, common 4 | - | 3 | Operating time, intraoperative blood loss, gastrointestinal function recovery time, hospital stay, complications, anastomotic leakage, intestinal obstruction, infection |
|  |  |  |  | TEPT | 15 | 9/6 | 28.8±15.6 | Long 4, short 6, common 5 |  |  |  |
| Tang | 2019 | China | Cohort | LEPT | 46 | 26/20 | 37.68±6.24 | Long 12, short 20, common 14 | 3 | 6 | Operating time, intraoperative blood loss, hospital stay, complications, HAEC, intestinal obstruction, infection |
|  |  |  |  | TEPT | 40 | 23/17 | 37.2±6.48 | Long 10, short 17, common 13 |  |  |  |
| Xie | 2019 | China | Retrospective cohort | LEPT | 44 | 32/12 | 47.04±21.12 | Long 12, short 12, common 20 | 6 | 6 | Operating time, intraoperative blood loss, gastrointestinal function recovery time, hospital stay, complications, HAEC, anastomotic leakage, intestinal obstruction, infection, soiling, constipation |
|  |  |  |  | OD | 44 | 29/15 | 49.44±22.44 | Long 15, short 11, common 18 |  |  |  |
| Xu | 2019 | China | Cohort | LEPT | 57 | 30/27 | 33.6 (8.4-72)* | - | - | 6 | Operating time, intraoperative blood loss, gastrointestinal function recovery time, hospital stay, complications, HAEC, anastomotic leakage, infection |
|  |  |  |  | TEPT | 56 | 29/27 | 28.8 (6-72)* |  |  |  |  |
| Zhang | 2019 | China | Cohort | LEPT | 27 | 14/13 | 8.5±0.5 | Total colonic 5, long 6, short 4, common 12 | - | 5 | Operating time, intraoperative blood loss, gastrointestinal function recovery time, complications, infection |
|  |  |  |  | OD | 26 | 13/13 | 8.5±0.7 | Total colonic 5, long 6, short 5, common 10 |  |  |  |
| Dai | 2018 | China | Retrospective cohort | LEPT | 26 | 14/12 | 42.6±9.6 | - | - | 5 | Intraoperative blood loss, gastrointestinal function recovery time, hospital stay, complications, HAEC, anastomotic leakage, infection, soiling, constipation |
|  |  |  |  | TEPT | 26 | 16/10 | 42.24±10.32 |  |  |  |  |
| Li | 2018 | China | Retrospective cohort | LEPT | 48 | 38/10 | 8.5±0.8 | Long 13, short 10, common 25 | 12 | 6 | Operating time, intraoperative blood loss, gastrointestinal function recovery time, hospital stay, complications, HAEC, anastomotic stricture, soiling, constipation |
|  |  |  |  | TEPT | 48 | 37/11 | 8.9±0.6 | Long 14, short 8, common 26 |  |  |  |
| Qin | 2018 | China | RCT | LEPT | 37 | 18/19 | 25.32±6.36 | Long 11, short 13, common 13 | - | 1 | Intraoperative blood loss, hospital stay, complications, intestinal obstruction, infection |
|  |  |  |  | TEPT | 37 | 20/17 | 24.96±6.24 | Long 12, short 14, common 11 |  |  |  |
| Sosnowska | 2018 | Poland | Retrospective cohort | TEPT | 22 | - | - | - | - | 3 | Soiling, constipation |
|  |  |  |  | OD | 9 |  |  |  |  |  |  |
| Zheng | 2018 | China | Retrospective cohort | LEPT | 78 | 60/18 | 11.5±12.7 | Ultrashort 10, rectosigmoid 35, descending colon 17, transverse colon 12, ascending colon 4 | 36-120 | 7 | Operating time, intraoperative blood loss, anastomotic strictures, intestinal obstruction, soiling, constipation |
|  |  |  |  | TEPT | 94 | 78/16 | 15.4±15.7 | Ultrashort 21, rectosigmoid 45, descending colon 24, transverse colon 4 |  |  |  |
| Deng | 2017 | China | RCT | LEPT | 22 | 13/9 | 33.72±8.88 | - | 3 | 3 | Operating time, intraoperative blood loss, gastrointestinal function recovery time, hospital stay, complications, HAEC, infection, soiling, constipation |
|  |  |  |  | TEPT | 20 | 12/8 | 34.68±8.52 |  |  |  |  |
| Fu | 2017 | China | RCT | TEPT | 59 | 33/26 | 14.17 ± 2.02 | Long 18, short 20, common 21 | - | 1 | Operating time, intraoperative blood loss, hospital stay, complications, HAEC, anastomotic stricture, intestinal obstruction, infection, soiling |
|  |  |  |  | OD | 59 | 31/28 | 14.52 ± 2.14 | Long 17, short 19, common 23 |  |  |  |
| Han | 2017 | China | Cohort | LEPT | 62 | 34/28 | 25.8±14.88 | Long 13, short 23, common 26 | 1 | 6 | Operating time, intraoperative blood loss, gastrointestinal function recovery time, hospital stay, complications, HAEC, anastomotic leakage, intestinal obstruction, infection |
|  |  |  |  | TEPT | 62 | 30/32 | 36.36±13.56 | Long 12, short 28, common 22 |  |  |  |
| He | 2017 | China | RCT | LEPT | 43 | 27/16 | 19.44±6.96 | Long 4, short 9, common 30 | 12 | 2 | Operating time, intraoperative blood loss, gastrointestinal function recovery time, hospital stay, complications, HAEC, anastomotic stricture, soiling, constipation |
|  |  |  |  | TEPT | 43 | 25/18 | 20.04±7.56 | Long 3, short 7, common 33 |  |  |  |
| Huang | 2017 | China | Retrospective cohort | LEPT | 60 | 38/22 | 3-54 | Rectosigmoid | 6 | 7 | Operating time, intraoperative blood loss, gastrointestinal function recovery time, complications, HAEC |
|  |  |  |  | TEPT | 60 | 40/20 | 3-60 |  |  |  |  |
| Guerra | 2016 | Canada | Retrospective cohort | LEPT | 24 | 16/8 | 3.5 ± 1.8 | Rectosigmoid | - | 5 | Operating time, hospital stay, complications, HAEC, anastomotic stricture, intestinal obstruction |
|  |  |  |  | TEPT | 12 | 7/5 | 4 ± 2.3 |  |  |  |  |
| Lukac | 2016 | Serbia | Retrospective cohort | TEPT | 30 | - | 9.41 ± 6.37 | Rectosigmoid | - | 5 | Complications, HAEC, anastomotic stricture |
|  |  |  |  | OD | 29 |  | - |  |  |  |  |
| Wang | 2015 | China | RCT | LD | 25 | 14/11 | 18.51±5.44 | Long 5, short 6, common 14 | - | 3 | Operating time, intraoperative blood loss, hospital stay, complications, HAEC, anastomotic leakage, intestinal obstruction, infection, constipation |
|  |  |  |  | OD | 25 | 15/10 | 18.01±5.42 | Long 6, short 7, common 12 |  |  |  |
| Yang | 2015 | China | Retrospective cohort | TEPT | 136 | 102/34 | 0.3-48 | Long 8, short 25, common 103 | - | 5 | Operating time, intraoperative blood loss, hospital stay, HAEC, anastomotic stricture, intestinal obstruction, infection, soiling, constipation |
|  |  |  |  | OD | 64 | 45/19 | 0.4-60 | Long 4, short 12, common 48 |  |  |  |
| Zhang | 2015 | China | Cohort | LEPT | 65 | 37/28 | 13.2±3.6 | Long 23, common 42 | - | 6 | Operating time, intraoperative blood loss, gastrointestinal function recovery time, complications, HAEC, intestinal obstruction, infection |
|  |  |  |  | OD | 63 | 35/28 | 14.4±2.4 | Long 22, common 41 |  |  |  |
| Zhou | 2014 | China | RCT | LEPT | 34 | 34/34 | 0.55±0.14 | Long 21, short 27, common 20 | - | 5 | Operating time, intraoperative blood loss, gastrointestinal function recovery time, hospital stay |
|  |  |  |  | OD | 34 |  |  |  |  |  |  |
| Chen | 2013 | China | Retrospective cohort | LEPT | 29 | 23/6 | 0.7-0.9 | Long 7, short 9, common 13 | 4-28 | 4 | Operating time, soiling |
|  |  |  |  | TEPT | 12 | 10/2 | 0.8-0.9 | Long 3, short 3, common 6 |  |  |  |
| Van de Ven | 2013 | Netherlands | Retrospective cohort | LEPT | 22 | 17/5 | 4.0 (1.5-43.8) | Rectosigmoid | 48 (10-71) | 7 | HAEC, anastomotic leakage, anastomotic stricture, intestinal obstruction, infection |
|  |  |  |  | TEPT | 21 | 17/4 | 2.4 (0.7-31.6) |  | 46 (24-76) |  |  |
| Wang | 2013 | China | Cohort | TEPT | 63 | - | - | - | 48-120 | 3 | Intestinal obstruction, soiling, constipation |
|  |  |  |  | OD | 7 |  |  |  |  |  |  |
| Chen | 2012 | China | Cohort | LEPT | 27 | 17/10 | 0.7-96 | Long 5, short 3, common 19 | - | 5 | Operating time, intraoperative blood loss |
|  |  |  |  | TEPT | 20 | 13/7 | 0.7-96 | - |  |  |  |
| Li_a | 2012 | China | RCT | LEPT | 30 | 22/8 | 26.4±2.2 | Long 8, common 52 | 12-48 | 1 | Operating time, hospital stay, intestinal obstruction, infection |
|  |  |  |  | OD | 30 | 24/6 | 25.8±1.9 |  |  |  |  |
| Li_b | 2012 | China | Retrospective cohort | LEPT | 42 | 148/26 | 34.08±3.84 | Long 5, common 37 | 6 | 6 | HAEC, anastomotic leakage, anastomotic stricture, intestinal obstruction, infection |
|  |  |  |  | TEPT | 82 |  |  | Short 13, common 69 |  |  |  |
|  |  |  |  | OD | 50 |  |  | Total colonic 2, long 4, common 44 |  |  |  |
| Nah | 2012 | UK | Retrospective cohort | LD | 35 | 29/6 | 3.1 (1.0-49.3) | Rectosigmoid | 80 (18-139) | 6 | Complications, HAEC, intestinal obstruction, soiling |
|  |  |  |  | OD | 41 | 32/9 | 3.3 (1.0-46.8) |  | 78 (12-130) |  |  |
| Dahal | 2011 | China | Retrospective cohort | LEPT | 33 | 27/6 | 17±18.2 | Long 12, common 21 | 29.4±16.2 | 5 | Soiling, constipation |
|  |  |  |  | TEPT | 98 | 85/13 | 13±8 | Long 6, common 92 |  |  |  |
| Giuliani | 2011 | Italy | Retrospective cohort | LD | 32 | 29/3 | 14.61 | Ultrashort 3, rectosigmoid 28, descending 1 | ≥12 | 4 | Complications, HAEC, anastomotic stricture, intestinal obstruction, constipation |
|  |  |  |  | OD | 24 | 21/3 | 13.28 | Ultrashort 2, rectosigmoid 15, descending 5, transverse-ascending 2 |  |  |  |
|  |  |  |  | LEPT | 14 | 12/2 | 4.67 | Ultrashort 2, rectosigmoid 10, descending 1, transverse-ascending 1 | 26 |  |  |
| Tang | 2011 | China | RCT | TEPT | 16 | 15/1 | 11.12±2.20 | - | - | 3 | Operating time, intraoperative blood loss, gastrointestinal function recovery time, hospital stay |
|  |  |  |  | OD | 18 | 16/2 | 24.23±3.02 |  |  |  |  |
| Wang | 2011 | China | Retrospective cohort | TEPT | 39 | 35/4 | 13.8±30.8 | Short or common | - | 6 | Operating time, intraoperative blood loss, hospital stay |
|  |  |  |  | OD | 39 | 32/7 | 20.0±29.4 |  |  |  |  |
| Gunnarsdottir | 2010 | Sweden | Cohort | TEPT | 11 | 7/4 | 4.8±5.2 | Rectosigmoid | 24 | 6 | Operating time, gastrointestinal function recovery time, hospital stay, HAEC, soiling, constipation |
|  |  |  |  | OD | 18 | 15/3 | 5.6±5.7 |  |  |  |  |
| Liu | 2009 | China | Retrospective cohort | LEPT | 23 | 13/10 | 38.4 (24-96)* | Short or common | 16 (3-48)* | 5 | Operating time, intraoperative blood loss, gastrointestinal function recovery time, anastomotic leakage, intestinal obstruction, infection, soiling, constipation |
|  |  |  |  | TEPT | 23 | 14/9 | 44.4 (24-108)* | Short 2, common 21 |  |  |  |
|  |  |  |  | OD | 23 | 14/9 | 45.6 (24-144)* | Short or common |  |  |  |
| Martins | 2009 | Brazil | Cohort | TEPT | 19 | 36/6 | 60 (12-108)* | - | - | 4 | Constipation |
|  |  |  |  | OD | 23 |  | 104 (24-180)* |  |  |  |  |
| Qin | 2009 | China | RCT | TEPT | 21 | 16/5 | 26.4±8.4 | Short 8, common 13 | 12-48 | 2 | Operating time, gastrointestinal function recovery time, hospital stay, complications, HAEC, anastomotic leakage, intestinal obstruction, infection, soiling, constipation |
|  |  |  |  | OD | 25 | 20/5 | 28.8±7.2 | Short 10, common 15 |  |  |  |
| Tannuri | 2009 | Brazil | Retrospective cohort | TEPT | 35 | - | 11.0 ± 15.1 | Rectosigmoid transition zone | 28.4 ± 20.6 | 4 | Operating time, hospital stay, HAEC, anastomotic stricture, infection, constipation |
|  |  |  |  | OD | 29 |  | 42.0 ± 34.8 |  | 60.5 ± 44.4 |  |  |
| Huang | 2008 | China | Retrospective cohort | LEPT | 29 | - | 16.8 (0.4-108)* | Short 11, common 18 | 12-60 | 5 | Operating time, hospital stay, HAEC, anastomotic leakage, intestinal obstruction, infection |
|  |  |  |  | TEPT | 44 |  |  | Short 14, common 30 |  |  |  |
|  |  |  |  | OD | 39 | 34/5 | 27.6 (3-120)* | Short 21, common 18 |  |  |  |
| Ishikawa | 2008 | Japan | Retrospective cohort | LEPT | 21 | - | 6.5±4.3 | - | 36 | 4 | Operating time, intraoperative blood loss, complications, HAEC, soiling, constipation |
|  |  |  |  | TEPT | 8 |  | 4.4±3.0 |  |  |  |  |
| Luo | 2007 | China | Cohort | LEPT | 24 | 34/13 | 1-132 | Long 4, short 15, common 28 | 6-48 | 5 | Infection, constipation |
|  |  |  |  | TEPT | 23 |  |  |  |  |  |  |
| Tang | 2007 | China | Retrospective cohort | LEPT | 70 | 49/21 | 34.8 (0.5-117.6) | Long 12, short 9, common 49 | 51 (3-91)* | 6 | Operating time |
|  |  |  |  | TEPT | 32 | 25/7 | 30 (0.4-64.8) | Short 10, common 22 |  |  |  |
| Travassos | 2007 | Netherlands | Retrospective cohort | LD | 30 | 23/7 | 8 (0.9–72) | - | 39.5 | 6 | HAEC, intestinal obstruction, constipation |
|  |  |  |  | OD | 25 | 21/4 | 6.8 (1.2–74.9) |  | 87.8 |  |  |
| Ren | 2005 | China | Retrospective cohort | TEPT | 54 | 48/6 | 6.4 (1-35)* | Short or common | 6±1 | 4 | Operating time, intraoperative blood loss, hospital stay, HAEC, anastomotic leakage, anastomotic stricture, intestinal obstruction, infection, constipation |
|  |  |  |  | OD | 54 | 48/6 | 5.5 |  |  |  |  |
| Wang | 2005 | China | Retrospective cohort | TEPT | 21 | 9/12 | 36 | Long 3, short 12, common 6 | 6-84 | 3 | Operating time, soiling |
|  |  |  |  | OD | 32 | 13/19 | 72 | Long 4, short 11, common 17 |  |  |  |

In the Author column, _a and _b were used to distinguish different studies; *mean (range); the units of age at surgery and follow-up time were months.

RCT, randomized controlled trial; M, male; F, female; HAEC, Hirschsprung-associated enterocolitis; OD, open Duhamel; LD, laparoscopic-assisted Duhamel; TEPT, transanal endorectal pull-through; LEPT, laparoscopic-assisted endorectal pull-through; LS, laparoscopic Soave.
